# Sequential Release of Paclitaxel and Imatinib from Core–Shell Microparticles Prepared by Coaxial Electrospray for Vaginal Therapy of Cervical Cancer

**DOI:** 10.3390/ijms22168760

**Published:** 2021-08-16

**Authors:** Zhepeng Liu, Haini Chen, Fengmei Lv, Jun Wang, Shoujin Zhao, Yijun Li, Xuexin Xue, Yu Liu, Gang Wei, Weiyue Lu

**Affiliations:** 1 School of Medical Instrument and Food Engineering, University of Shanghai for Science and Technology, Shanghai 200093, China; 193832383@st.usst.edu.cn (H.C.); 181700124@st.usst.edu.cn (F.L.); 20111030011@fudan.edu.cn (J.W.); 183852335@st.usst.edu.cn (S.Z.); 172702210@st.usst.edu.cn (Y.L.); 182702178@st.usst.edu.cn (X.X.); 2 Department of Pharmaceutics, School of Pharmacy, Fudan University & Key Laboratory of Smart Drug Delivery (Fudan University), Shanghai 201203, China; weigang@shmu.edu.cn (G.W.); wylu@shmu.edu.cn (W.L.)

**Keywords:** coaxial electrospray, core–shell microparticles, sequential release, cervical cancer, paclitaxel, imatinib

## Abstract

To optimize the anti-tumor efficacy of combination therapy with paclitaxel (PTX) and imatinib (IMN), we used coaxial electrospray to prepare sequential-release core–shell microparticles composed of a PTX-loaded sodium hyaluronate outer layer and an IMN-loaded PLGA core. The morphology, size distribution, drug loading, differential scanning calorimetry (DSC), Fourier transform infrared spectra (FTIR), in vitro release, PLGA degradation, cellular growth inhibition, in vivo vaginal retention, anti-tumor efficacy, and local irritation in a murine orthotopic cervicovaginal tumor model after vaginal administration were characterized. The results show that such core–shell microparticles were of spherical appearance, with an average size of 14.65 μm and a significant drug-loading ratio (2.36% for PTX, 19.5% for IMN, *w*/*w*), which might benefit cytotoxicity against cervical-cancer-related TC-1 cells. The DSC curves indicate changes in the phase state of PTX and IMN after encapsulation in microparticles. The FTIR spectra show that drug and excipients are compatible with each other. The release profiles show sequential characteristics in that PTX was almost completely released in 1 h and IMN was continuously released for 7 days. These core–shell microparticles showed synergistic inhibition in the growth of TC-1 cells. Such microparticles exhibited prolonged intravaginal residence, a >90% tumor inhibitory rate, and minimal mucosal irritation after intravaginal administration. All results suggest that such microparticles potentially provide a non-invasive local chemotherapeutic delivery system for the treatment of cervical cancer by the sequential release of PTX and IMN.

## 1. Introduction

Combination therapy is widely used in clinical practice [1,2,3,4]. Many micron/nano preparations have been designed for the co-administration of different anti-tumor drugs [5,6,7]. However, the simple simultaneous administration of two or more drugs may not obtain satisfactory clinical outcomes, mainly because of unfavorable drug–drug interactions, differences in pharmacokinetics, and unsynchronized biodistribution [8,9,10,11]. 

In comparison to simultaneous administration, formulations that can precisely release different drugs in a controlled and, more specifically, sequential manner can provide maximized therapeutic efficacy and reduced adverse effects by minimizing drug–drug interactions, reducing side effects that may be associated with simultaneous co-delivery, and overcoming anticancer drug resistance [12,13,14,15,16,17]. Hu et al. incorporated PTX nanoparticles and lapatinib microparticles into a thermosensitive hydrogel to achieve a short-term release of PTX and a long-term release of lapatinib [16]. Zhang et al. prepared dexamethasone/docetaxel-co-loaded liposomes with the sequential release properties of a quick dexamethasone release and a prolonged docetaxel release [13]. Recently, Sui et al. tested a sequential administration schedule of sialic-acid-modified epirubicin liposome followed by sialic acid-modified zoledronate liposomes and reported total tumor growth inhibition [17]. However, a scaled-up production of these preparations constituted challenges due to complex processing [18,19,20,21]. 

Electrospray might be a suitable alternative strategy for preparing sequential-release formulations [22], as coaxial electrospray can build fine multi-layer structures [23]. There have been many reports of core–shell nanofibers and nanotubes built by coaxial electrospinning. Yang et al. prepared a drug delivery system with a tunable zero-order release characteristic by triaxial electrospinning. Zandi et al. fabricated core–shell fibers for the co-delivery of lysozyme and phenytoin sodium by coaxial electrospinning for tissue injury treatment [24,25,26,27]. However, coaxial electrospray has seldom been reported for the preparation of core–shell micro/nanoparticles.

Paclitaxel (PTX), a typical tubulin inhibitor that induces mitotic stagnation of tumor cells in the G2 and M stages, has been widely studied in the treatment of cervical and breast cancers [28]. However, resistance to PTX is frequently observed, and is possibly related to an overexpression of P-gp efflux pumps, multidrug resistance (MDR-1) gene, and a high expression of a growth factor, such as platelet-derived growth factor (PDGF) [29,30,31]. Imatinib (IMN), the first tyrosine kinase inhibitor approved for the treatment of chronic myelogenous leukemia and malignant gastrointestinal stromal tumor [32,33], has been reported to inhibit the growth of cervical cancer cells by inhibition of PDGFR and c-kit [34,35]. The combination of PTX with IMN has shown good clinical potential in trials [36,37]. 

**Figure 1:**
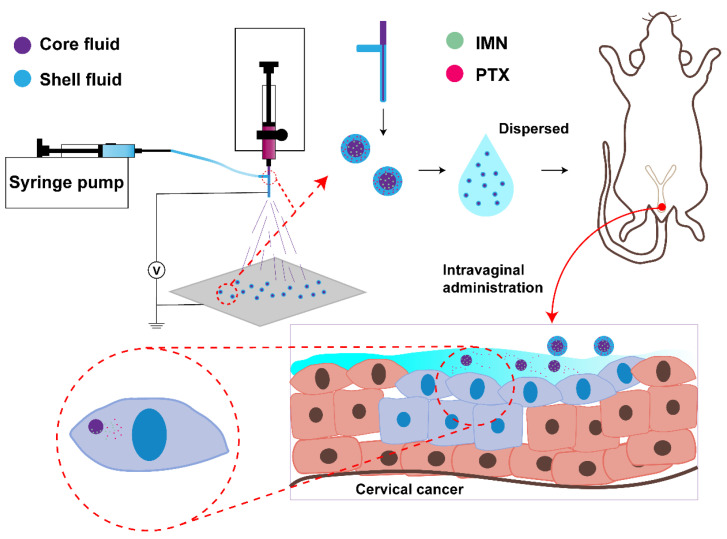
Illustration of the preparation, by coaxial electrospray, of the core–shell microparticle and its action mechanism.

In our study, to improve antitumor effects and reduce side effects, sequential-release core–shell microparticles were designed by coaxial electrospray (Figure 1), fabricated with a PTX-loaded hyaluronate (HA) outer layer and an IMN PLGA core. This core–shell microparticle was expected to first release the PTX quickly and, subsequently, release the IMN slowly, which acted upon the highly expressed PDGF induced by the treatment with PTX. This core–shell microparticle is for vaginal administration in the treatment of cervical cancer, which is a serious concern for women and caused approximately 25,000 deaths in 16 countries in 2015 [38]. The HA shell material has appropriate biocompatibility, mucoadhesion, and intra-tumor permeability [39,40]. Vaginal application is beneficial for the treatment of cervical cancers because the drug can be delivered to the tumor site in a direct and controlled manner [41,42]. 

To the best of our knowledge, this is the first report on micron preparations fabricated by electrospray for sequential-release anti-tumor drugs. The morphology, size distribution, drug loading, encapsulation efficiency, differential scanning calorimetry (DSC), Fourier transform infrared spectra (FTIR), in vitro release, degradation of microparticles, cellular growth inhibition, in vivo vaginal retention, anti-tumor efficacy, and local irritation in a murine orthotopic cervicovaginal tumor model after vaginal administration were characterized.

## 2. Results and Discussion 

### 2.1. Preparation and Characterization of Microparticles

**Figure 2:**
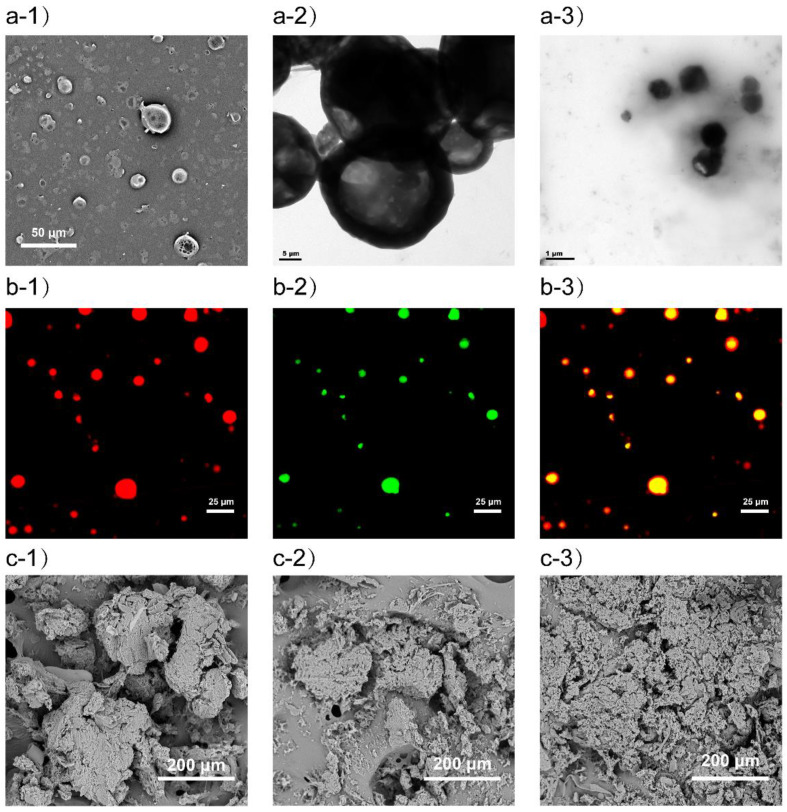
Morphology of core–shell microparticles composed of the PTX-loaded sodium hyaluronate outer layer and the IMN-loaded PLGA core (PHIPMPs): (**a-1**) TEM morphology of PHIPMPs; (**a-2**) TEM morphology of PHIPMPs incubated in a pH = 7.4-PBS solution for 5 min; (**a-3**) TEM morphology of PHIPMPs incubated in pH7.4 PBS solution for 1 h; (**b-1**–**b-3**) Confocal microscopy image of fluorescent-labeled PHIPMPs; (**b-1**) RhB, labeling the shell; (**b-2**) coumarin-6, labeling the core; (**b-3**) merged; (**c-1**–**c-3**) SEM morphology of PHIPMPs incubated in pH7.4 PBS for 3, 5, and 7 days, respectively.

**Figure 3:**
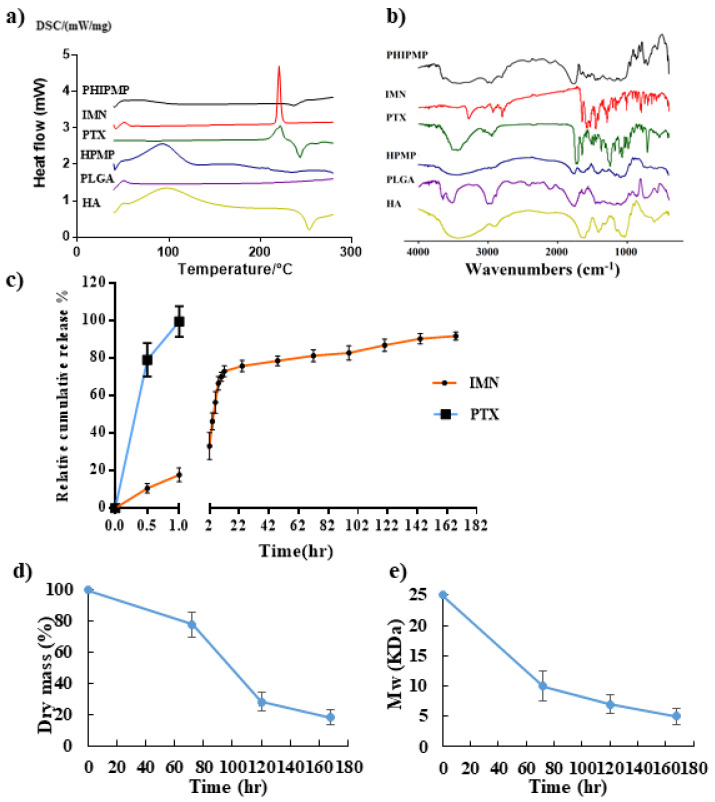
Characteristics of PHIPMPs: (**a**) DSC; (**b**) FTIR; (**c**) in vitro release of IMN and PTX in PHIPMPs; (**d**) weight change in PHIPMPs; (**e**) average molecular weight change of PHIPMPs (*n* = 3, mean ± SD).

By means of coaxial electrospray, drugs can be conveniently loaded in different areas of microparticles to achieve precise control of the drug release [43,44,45]. As shown in Figure 1, PTX/HA microparticles containing IMN/PLGA nanoparticles (PHIPMPs) were of near-spherical morphology (Figure 2a-1). Figure 2a-2 shows that PHIPMPs were dispersed in saline solution after 5 min. The mean size of the PHIPMPs shown by DLS was about 14.65 μm. Figure 2a-3 shows that the PHIPMPs collapsed to nanoparticles after dispersion in the saline solution for 1 h. There was red fluorescence in the shell (Figure 2b-1) and green fluorescence in the core (Figure 2b-2). Figure 2b-3 shows the core (green) was wrapped in an outer layer (red), confirming the core–shell structure of the PHIPMPs. The DL of PTX and IMN in the PHIPMPs were 2.36% and 19.5%, respectively. The EE of PTX and IMN were 94.4% and 97.5%, respectively. 

Thermal analysis has been widely applied to investigate the drug–carrier interactions and may reveal possible changes to the physical state. As shown in Figure 3a, the thermogram of bulk PTX and IMN displayed an exothermic peak at about 222 °C and 213 °C, respectively, which was not observed in the PHIPMPs, indicating a certain change in the physical state of PTX and IMN after encapsulation in PHIPMPs. The state of the drug in PHIPMPs may be an amorphous, disordered crystalline phase or a solid solution state. 

FTIR spectra of PTX, IMN, PLGA, HA, HPMPs, and PHIPMPs are displayed in Figure 3b. The spectra of the PHIPMPs show characteristic peaks at 2831 cm^−1^ (v C–H) and 1657 cm^−1^ (v C=O) for IMN, as well as at 1603 cm^−1^ (v C=C) for PTX [46,47]. There are characteristic peaks of PLGA and HA, but no formation of new peaks in the spectra of the PHIPMPs. Characteristic peaks of PTX are unclear, perhaps due to a low PTX content loaded in the microparticles. Compared to the spectra of the PHIPMPs and IMN, the spectra of the PHIPMPs provided information about the substantial decrease in the intensities of characteristic peaks and peaks in the finger regions of IMN. The decrease might be attributed to a hydrophobic interaction between the long carbon chain of PLGA and the benzene ring of IMN, and is perhaps beneficial for the compatibility between the drug and carrier material and, thus, the stability of the obtained microparticles.

### 2.2. In Vitro Release of PTX and IMN

The in vitro release behavior of IMN and PTX from PHIPMPs in VFS (pH = 4.2) is shown in Figure 3c. The PTX was immediately released (about 80% at 0.5 h and nearly 100% at 1 h), while the release of IMN was sustained (<20% at 1 h and nearly 90% on day 7), indicating the successful realization of the sequence release of PTX and IMN from PHIPMPs, which might be of significance for strengthening the synergistic effect of the drug combination [15]. In the present study, PTX and IMN were co-loaded into the same core–shell microparticles in one-step by electrospray, realizing the purpose of the sequential release of PTX and IMN.

### 2.3. Degradation of PHIPMPs

The degradation behavior of the PHIPMPs was monitored by the change in weight, average molecular weight, and surface morphology (Figure 2c-1–c-3, Figure 3d,e). The tendencies in the changes in weight and average molecular weights were similar. The PHIPMPs underwent degradation with the elapse of time. The degradation of the PLGA could be divided into two stages—an initial rapid degradation within the first 3 days (in which the average molecular weight was between 25,000 and 9700), and a gradual decrease in the subsequent 7 days. The sample was fragmented after 7 days of degradation. Consistent with the dissolution data, the IMN in the PHIPMPs was completely released within 7 days. 

### 2.4. Cytotoxicity Assay

PTX and IMN showed good synergistic effects on TC-1 cells. The IC_50_ of the PTX/IMN physical mixture (1:8) was 2.624 μM, much lower than that of free PTX (51.67 μM) and IMN (21.79 μM). However, the PHIPMPs showed the strongest synergy effect, as the CI was 0.09 (the CI of the PTX/IMN physical mixture was 0.64), indicating that the combination of PTX and IMN in the form of a core–shell microparticle can exert a better synergistic effect. The PHIPMPs showed stronger cytotoxic effects in the experiment, which might be attributed to the sequential drug release. IMN inhibited highly expressed PDGF induced by PTX treatment [35].

### 2.5. Vaginal Retention

**Figure 4:**
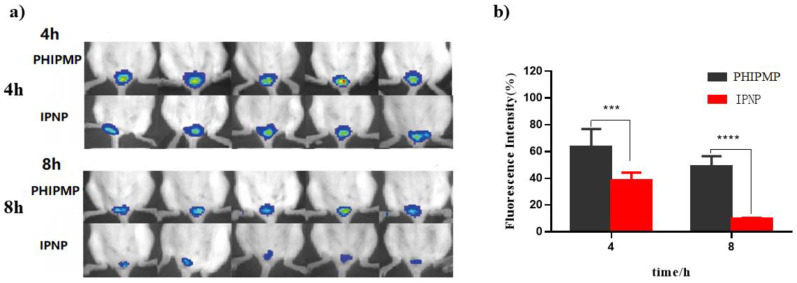
Intravaginal retention of DiR-labeled formulations 4 and 8 h after intravaginal administration. (**a**) Typical fluorescent images; (**b**) semiquantitative data of the fluorescence intensity (*n* = 5), *** indicates *p* < 0.001, **** indicates *p* < 0.0001. (PHIPMP: PTX/HA-IMN/PLGA microparticles; IPNP: IMN/PLGA nanoparticles).

Imaging of fluorescent-labeled PHIPMPs and IPNPs in the vagina at 4 and 8 h is shown in Figure 4a. The fluorescent imaging at hours 1–24 is provided in Figure S2 (see Supplementary Materials). The fluorescence of the PHIPMPs remained in the vagina for up to eight hours after administration. The semiquantitative fluorescence of the PHIPMPs was significantly higher at hour 4 and 8 compared to the IPNPs (63.70% ± 13.18% vs. 37.92% ± 5.26% and 48.72% ± 7.84% vs. 10.05% ± 0.34%, respectively, Figure 4b), indicating prolonged intravaginal residence. The retention time of the PHIPMPs in the vaginal mucosa was prolonged, perhaps benefitting from the adhesion effect of HA. 

### 2.6. In Vivo Antitumor Study 

**Figure 5:**
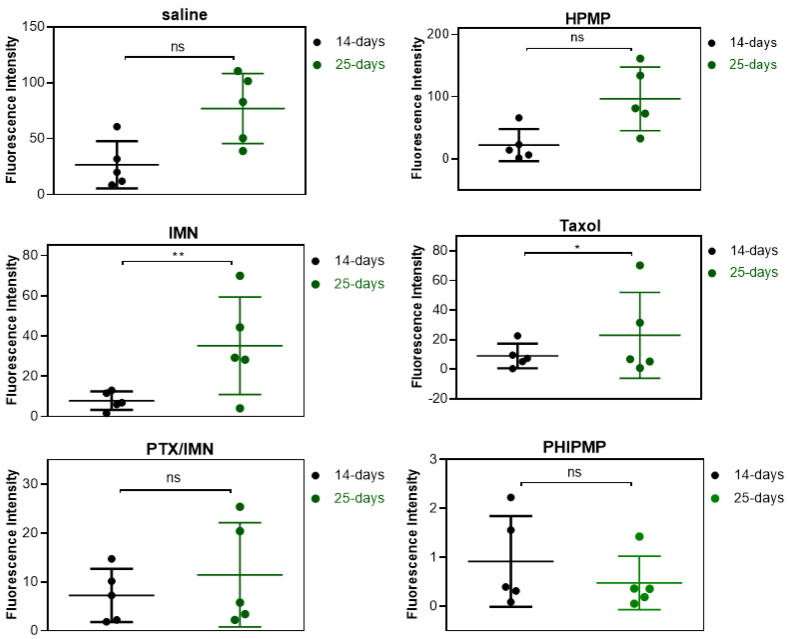
Bioluminescence of each group relative to the tumor-bearing mice on the 14th and 25th days (● the 14th day, ● the 25th day (*n* = 5).

**Figure 6:**
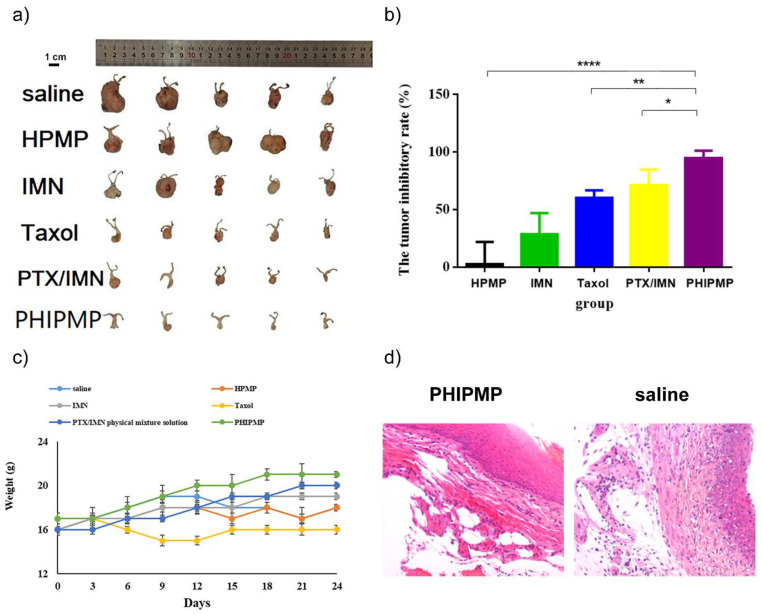
In vivo pharmacological evaluation. (**a**) The tumor size of each group after treatment; (**b**) tumor inhibitory rate (*n* = 5); (**c**) body weight change (*n* = 5); (**d**) vagina sections stained with H&E. * indicates *p* < 0.05, ** indicates *p* < 0.01, **** indicates *p* < 0.0001. (HPMP: HA/PLGA microparticles; IMN: Imatinib; PTX: Paclitaxel; PHIPMP: PTX/HA-IMN/PLGA microparticles).

Figure S3 (Supplementary Materials) showed the images of tumor-bearing mice treated with PHIPMPs, PTX/IMN physical mixture solution, Taxol^®^, IMN solution, HPMPs, and saline. The fluorescence intensity indicated the tumor size. The fluorescence intensity of each group on the 14th and 25th days is shown in Figure 5. The fluorescence intensity of the IMN solution group and Taxol^®^ group increased significantly on the 25th day compared to the 14th day (*p* < 0.05). By contrast, the PHIPMP group showed no significant increase in fluorescence intensity from the 14th to the 25th days, which is in accordance with the final anatomical observation (Figure 6a). 

Figure 6b shows the calculated tumor inhibitory rate of all tested groups. There was no therapeutic effect in the HPMP group. The PHIPMP group showed the greatest anti-tumor effect (*p* < 0.05). Tumor anatomical observation showed that the tumors almost disappeared after treatment with PHIPMPs, with a tumor inhibitory rate exceeding 90%, which was significantly higher than that of the PTX/IMN mixture at the same dosage, exhibiting the advantage of sequential drug release. The effect on the PTX/IMN physical mixing solution group was lower than that of the PHIPMP group, perhaps because the drugs were quickly eliminated in this group. As shown in Figure 6c, the body weight of the Taxol^®^ group (3.6 mg/kg) was lower than those of the other groups, indicating greater toxicity. In contrast, the body weight of the animals in the PHIPMP group increased.

As shown in Figure 6d, the morphology of the vagina mucosa in the PHIPMP group was similar to that in the blank group, showing no irritation. The morphology of the liver, heart, kidney, lung, and spleen showed no significant change in the PHIPMP group in comparison with the blank group (Figure S4, Supplementary Materials).

## 3. Materials and Methods

### 3.1. Materials

PLGA (50:50, *M*_W_ = 25,000) was obtained from Jinan Daigang Biomaterial Co., Ltd. (Jinan, China). Sodium hyaluronate (HA) was purchased from Bloomage Biotechnology Co., Ltd. (*M*_W_ = 800 kDa) (Jinan, China). Paclitaxel (PTX) and imatinib (IMN) were purchased from Dalian Meilun Biotechnology Co., Ltd. (Dalian, China) and Shanghai McLean Biochemical Technology Co., Ltd. (Shanghai, China), respectively. Potassium (S)-2-(6-hydroxybenzo[d]thiazol-2-yl)-4,5-dihydrothiazole-4-carboxylate (d-luciferin potassium salt, DLPS), FITC, RhB, DiR, and coumarin-6 were purchased from Dalian Meilun Biotechnology Co., Ltd. (Dalian, China). Other chemicals and solvents were purchased from Sinopharm Chemical Reagent Co., Ltd. (Shanghai, China). 

A cervical-cancer-related TC-1 cell line was obtained from the Cell Bank of the Chinese Academy of Sciences. ICR and C57 mice (female, 6–8 weeks old) were from Shanghai Sipur-Bikai Experimental Animal Co., Ltd. (Shanghai, China) and kept under SPF conditions throughout the experiments. All studies were performed with the approval of the Institutional Animal Care and Use Committee of Fudan University.

### 3.2. Fabrication of the PTX/HA-IMN/PLGA Microparticles (PHIPMPs), IMN/PLGA Nanoparticles (IPNP), and HA/PLGA Microparticles (HPMPs)

PTX/HA–IMN/PLGA microparticles (PHIPMPs) were electrosprayed using a co-axial needle. First, an IMN and PLGA mixed solution was prepared by dissolving IMN (32 mg) and PLGA (120 mg) in 10 mL of dichloromethane, and then a PTX HA solution was prepared by dissolving PTX (2 mg) and HA (2 mg) in 10 mL of an ethanol/water mixture (1:3, *v*/*v*). Each solution was fed into two 10 mL plastic syringes connected to a co-axial needle, which was controlled by a syringe pump with a mass flow rate of 1 mL/h for the inner core (IMN/PLGA solution) and of 2 mL/h for the outside chamber (PTX/HA solution). A voltage of 17 KV was applied. The microparticles were collected on a glass slide with a nozzle-collector at a distance of 15 cm. Fluorescent-labeled PHIPMPs were prepared using the same method, except FITC or RhB was added to the outer layer and Dir or coumarin-6 was added to the inner layer. IMN/PLGA nanoparticles (IPNPs) were electrosprayed using the same equipment and process condition, except the outside chamber flow rate was 0 (PTX/HA solution). HA/PLGA microparticles (HPMPs) were electrosprayed using the same method, except no PTX or IMN was added to the outer or inner solution.

### 3.3. Structural Morphology and Size Distribution

The morphology of the PHIPMPs was observed by scanning electron microscopy (SEM) with an accelerating voltage of 15 kV. Before observation, the PHIPMPs were coated with gold using a sputter coater. The PHIPMPs were dispersed in a saline solution and then observed by transmission electron microscope (TEM) after 5 min and again after 1 h. Fluorescent-labeled PHIPMPs were observed by laser scanning confocal microscopy (TCS SP8, Leica, German) using excitation spectra of 550 nm (for RhB, red color, for the shell) and 466 nm (for coumarin-6, green color, for the core). The particle size distribution of the PHIPMPs was determined by dynamic laser scattering (DLS, Mastersizer 2000, Malvern, U.K.) at 25 °C.

### 3.4. Drug Loading (DL) and Encapsulation Efficiency (EE)

The drug loading (DL) and encapsulation efficiency (EE) of the PTX and IMN in the PHIPMPs were analyzed by high-performance liquid chromatography (HPLC, Waters 2695, equipped with a binary pump and PDA detector). The chromatographic conditions comprised an Xtimate^®^ C18 column (25 cm × 4.6 mm, particle size: 5 μm, Welch Technology, Shanghai, China), acetonitrile as mobile phase A, and 0.1% phosphoric acid solution as mobile phase B. The gradient elution conditions are provided in Table S1 (Supplementary Materials). The injection volume was 20 μL, the column temperature was 30 °C, the flow rate was 1.0 mL/min, and the detection UV wavelength was 254 nm. 

Lyophilized PHIPMPs were accurately weighed and dissolved in 80% acetonitrile for HPLC analysis. The retention time of PTX and IMN was about 2.3 and 8.2 min, respectively (Figure S1, see Supplementary Materials). The external standard method was used to quantify the contents of PTX and IMN by integrating the areas under the peaks. Calibration curves were performed in a concentration range of 40–300 μg/mL with IMN and 10–60 μg/mL with PTX. 

The DL and EE of the PTX and IMN were calculated using the following equations:

DL(%)=(W PTX or IMN in PHIPMP/Wtotal PHIPMP)×100%

EE(%)=(W encapsulated PTX or IMN/W input PTX or IMN)×100%

### 3.5. Physical Characterization

Differential scanning calorimeter (DSC) analysis was carried out with a PerkinElmer Pyris 1 DSC instrument (Waltham, MA, USA) equipped with an Intra-cooler 2P cooling accessory. The samples were accurately weighed, separately sealed in standard aluminum pans, and scanned from 25 to 280 °C with a heating rate of 10 °C/min and a nitrogen purge of 10 mL/min.

The PTX, IMN, PLGA, HA, HPMPs, and PHIPMPs were characterized by a Fourier transform infrared spectrometer (FTIR, AVATAR 360) at a 4 cm^−1^ resolution in a range of 400–4000 cm^−1^. 

### 3.6. In Vitro Release 

The dialysis method was used to evaluate the in vitro drug release behavior [48]. First, PHIPMPs (5 mg) were placed in a dialysis bag (with a molecular weight cut-off of 3500 Da), which was placed in 3 mL of VFS solution (pH = 4.2, containing 0.1% Tween 80) as the release media and shaken at 100 rpm at 37 °C for one week. At pre-determined time points (0.5, 1, 2, 4, 6, 8, 10, 12, 24, 48, 72, 96, 120, 144, and 168 h), the release medium was totally replaced by pre-warmed fresh medium. The release media samples were submitted to HPLC determination using similar conditions as described in Section 2.4. 

### 3.7. Degradation of PHIPMPs

PHIPMPs were placed in dialysis bags (with a molecular weight cut-off of 3500 Da), which were placed in 3 mL of PBS solution (pH = 7.4) as the release media and shaken at 100 rpm at 37 °C. At pre-determined time points (72, 120, and 168 h), the contents in the dialysis bags were removed, washed three times with water (1 mL each time), and freeze-dried. They were then submitted for SEM observation and GPC analysis for molecular weight measurement. GPC was performed on an Agilent system, with a 2695 Refractive Index detector. Two PL-gel 5 μm mixed-D columns fitted with guard columns (Polymer Labs, molecular weight range of 0.2–400 kDa) were used. THF (1 mL/min) was used as a mobile phase. Calibration was performed with polystyrene standards.

### 3.8. Cellular Experiments

TC-1 cells were cultured in DMEM containing 10% fetal bovine serum (FBS) with 10,000 units/mL of penicillin and 10,000 μg/mL of streptomycin at 37 °C in a humidified atmosphere containing 5% CO_2_. They were left to differentiate for 14 days prior to the experiments. The medium was replaced every 2–3 days.

The cytotoxicity of free PTX, free IMN, the PTX/IMN physical mixture, and PHIPMPs against TC-1 cells were compared by a standard MTT method [49]. The plates were read at 490 nm with a BioTek Power Wave XS reader (Power Wave XS, BioTek, Winooski, VT, USA). Each tested concentration was replicated six times. The IC_50_ was calculated with a GraphPad Prism 6 (La Jolla, CA, USA). The combined index (CI) was calculated as follows:

$$\mathrm{CI}=\frac{D_{A}}{{\mathrm{IC}}_{50,A}}+\frac{D_{B}}{{\mathrm{IC}}_{50,B}}$$

where A and B represent drugs A and B, respectively; IC_50,A_ and IC_50,B_ are the IC_50_ values of A and B, respectively (used separately); and D_A_ and D_B_ are the IC_50_ values of A and B (used together).

### 3.9. Vaginal Retention Experiment

Fluorescent-labeled PHIPMPs and IMN/PLGA nanoparticles (IPNPs) were intravaginally administered to healthy ICR female mice (20–25 g). Imaging of the fluorescence intensity was observed by FCM analysis (BD FACSCalibur, Jersey City, NJ, USA) at pre-determined time points (1, 2, 4, 8, 12, and 24 h). 

### 3.10. In Vivo Antitumor Study

An animal model of cervical cancer was established in mice. First, 30 C57 female mice were randomly divided into 6 groups (*n* = 5). After peritoneal anesthesia (10 μL of chloral hydrate), the vaginas of the mice were everted with hemostatic forceps, the vaginal mucosa was scraped with a scalpel and TC-1 cells (10^5^/10 μL) were injected [35,50]. Three days after inoculation, the mice were intraperitoneally injected with d-fluorescein potassium salt for imaging in vivo. The tumor imaging of fluorescence intensity was observed by FCM analysis (BD FACSCalibur, Jersey City, NJ, USA).

PHIPMPs, the PTX/IMN physical mixture solution, Taxol^®^, IMN solution, HPMPs, and saline were intravaginally administered to the mice on the 4th, 9th, and 14th days, with doses of PTX 1 mg/kg and IMN 8 mg/kg, and a dose of 3.6 mg/kg for the Taxol^®^ group. The animals were weighed every three days. On the 4th, 14th, and 25th days, the tumor sizes were measured by fluorescence intensity by FCM analysis (BD FACSCalibur, Jersey City, NJ, USA). The relative bioluminescence of each group was calculated as the fluorescence intensity on the 14th and 25th days divided by the fluorescence intensity on the 4th day after inoculation. The mice were sacrificed by cervical vertebra dislocation on the 25th day to determine the tumor weights. The tumor inhibitory rate for each treatment group was calculated as follows:

Tumor inhibitory rate=(Tumor Weight treatment group/Tumor Weight saline group)×100%

The vagina, heart, lung, spleen, liver, and kidney tissues of the PHIPMPs treatment group and the blank group were fixed in 10% formalin, embedded in paraffin, sectioned at a thickness of 4 μm, and stained with hematoxylin and eosin (H&E). 

### 3.11. Statistical Analysis

All data are expressed as the mean value ± SD. Statistical analysis was performed with one-way analysis of variance (ANOVA) in Graph Pad Prism 6 software. A *p*-value of less than 0.05 is considered statistically significant.

## 4. Conclusions

From the coaxial electrosprayed core–shell microparticles composed of a PTX-loaded sodium hyaluronate outer layer and an IMN-loaded PLGA core, the PTX was rapidly released from the shell, and the IMN was subsequently released slowly from the PLGA core, achieving good in vitro and in vivo therapeutic effects. Such microparticles might provide a potentially non-invasive local chemotherapeutic delivery system for future treatment of cervical cancer.

## Data Availability

Not applicable.

## Supplementary Materials

The following are available online at https://www.mdpi.com/article/10.3390/ijms22168760/s1.
