# Supplementary material for: Sequential Release of Paclitaxel and Imatinib from Core–Shell Microparticles Prepared by Coaxial Electrospray for Vaginal Therapy of Cervical Cancer
Source: Int J Mol Sci. 2021 Aug 16;22(16):8760. doi: 10.3390/ijms22168760 (PMC8395827; doi:10.3390/ijms22168760)
Supplement: Supplementary file 1 [file ijms-22-08760-s001.zip › ijms-1304528-supplementary.pdf]

Sequential release of paclitaxel and imatinib from core-shell microparticles prepared by coaxial electrospray for vaginal therapy of cervical cancer

Zhepeng Liu[a*], Haini Chen[a], Fengmei Lv[a], Jun Wang[a,b], Shoujin Zhao[a], Yijun Li[a], Xuexin Xue[a], Yu Liu[b*], GangWei[b], Weiyue Lu[b]

[a]*School of Medical Instrument and Food Engineering, University of Shanghai for Science and Technology, Shanghai 200093, China*

[b]*Department of Pharmaceutics, School of Pharmacy, Fudan University & Key Laboratory of Smart Drug Delivery (Fudan University), Shanghai 201203, China*

Table S1. Gradient elution for HPLC analysis

Time (min)	Mobile phase A (per cent V/V)	Mobile phase B (per cent V/V)
0-5	100	0
5-15	70	30
15-23	100	0

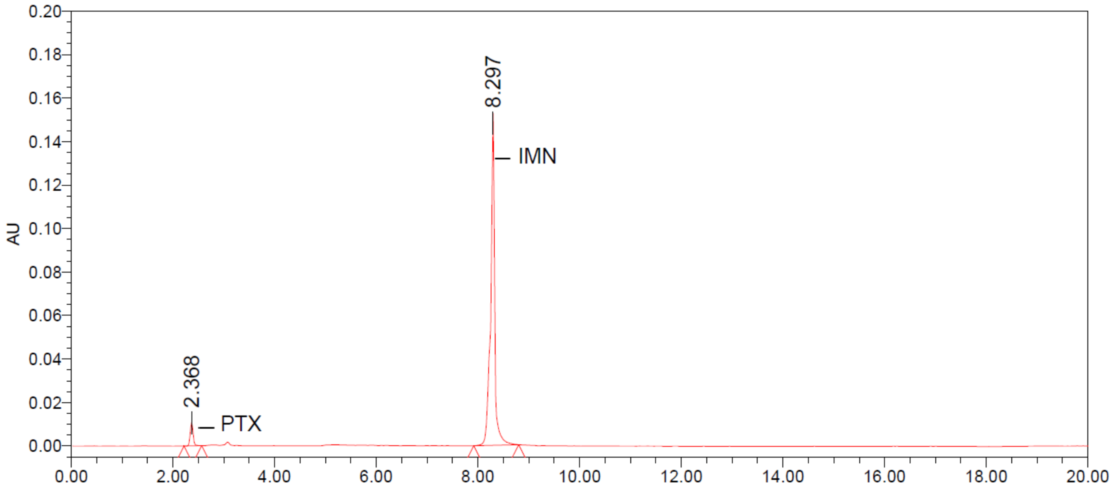

Fig.S1 The HPLC of PTX and IMN.

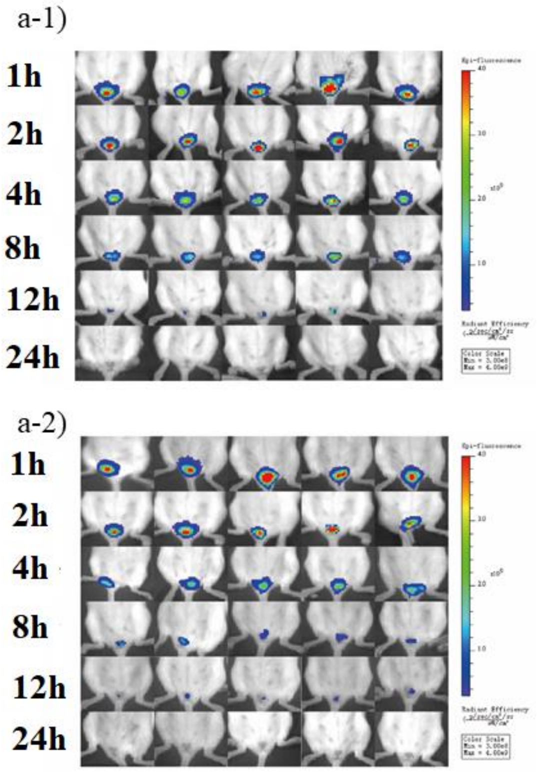

Fig.S2 The fluorescence comparison of PHIPMP and IPNP in vaginal at 1, 2, 4, 8 and 12 hours (a-1. PHIPMP; a-2. IPNP).

a) the 4th day

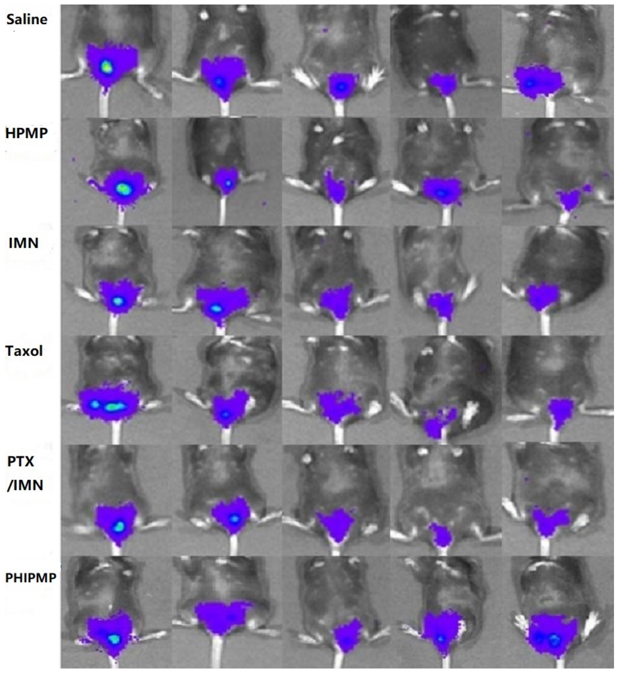

b) the 14th day

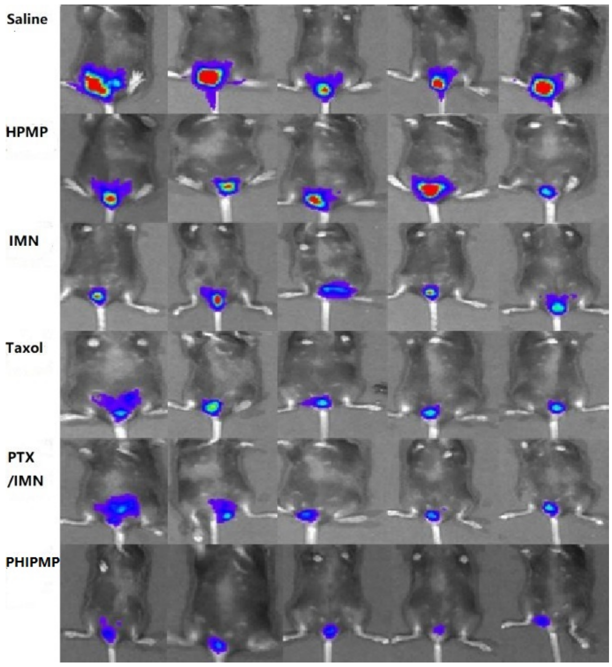

c) the 25th day

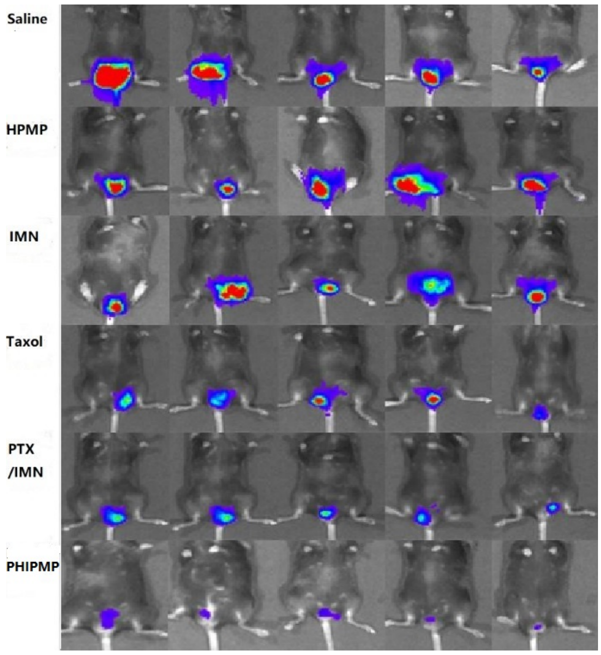

Fig.S3 The bioluminescence intensity imaging of PHIPMP, PTX/IMN physical mixture solution, Taxol, IMN solution, HPMP and saline group to the tumor-bearing mice at the 4th, 14th and 25th day (n=5).

a)

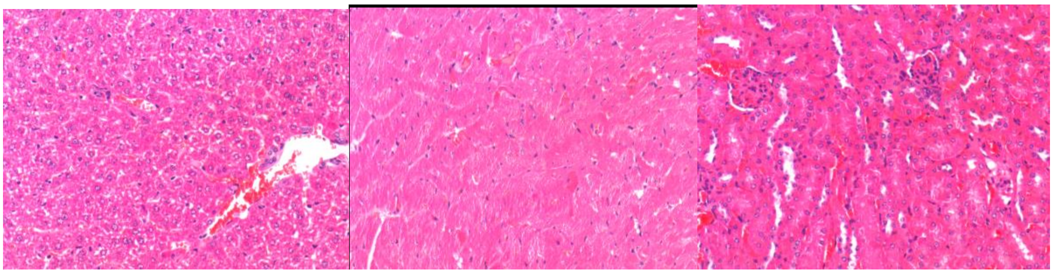

liver heart kidney

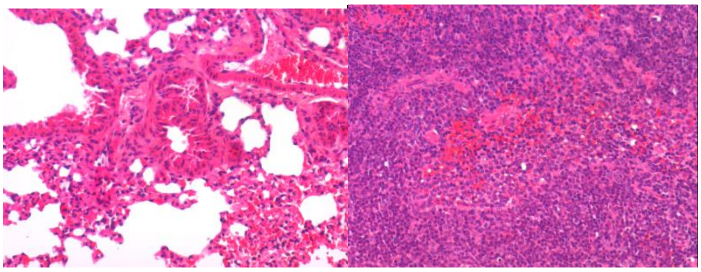

lung spleen

b)

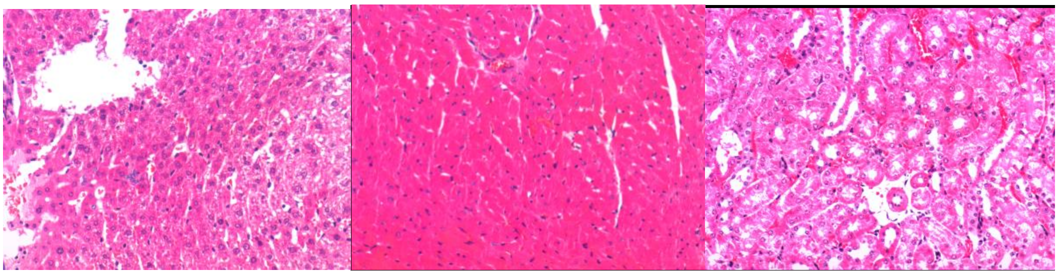

liver heart kidney

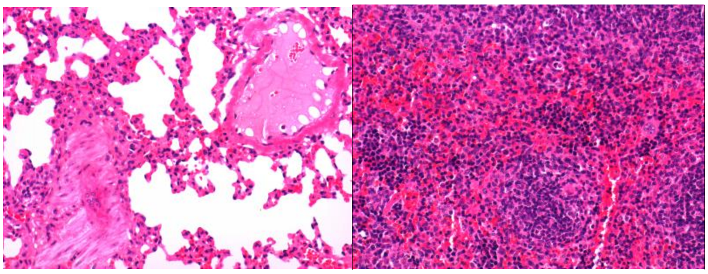

lung spleen

Fig. S4 Tissues stained with H&E of liver, heart, kidney, lung and spleen (a. PHIPMP; b.saline)
